# Association between the chronology of gestation and the morphometrical skin characteristics at childbirth: a development of predictive model

**DOI:** 10.1136/bmjhci-2021-100476

**Published:** 2021-12-07

**Authors:** Ingrid Michelle Fonseca de Souza, Gabriela Luiza Nogueira Vitral, Marcelo Vidigal Caliari, Zilma Silveira Nogueira Reis

**Affiliations:** 1 Obstetrics and Gynecology, Universidade Federal de Minas Gerais, Belo Horizonte, Minas Gerais, Brazil; 2 Pediatrics, Postgraduation Program, Universidade Federal de Minas Gerais, Belo Horizonte, Minas Gerais, Brazil; 3 Department of General Pathology, Universidade Federal de Minas Gerais, Belo Horizonte, Minas Gerais, Brazil; 4 Informatics Health Center, Universidade Federal de Minas Gerais, Belo Horizonte, Minas Gerais, Brazil

**Keywords:** delivery of health care, image processing, computer, Probability, medical informatics applications

## Abstract

**Objective:**

The structural maturation of the skin is considered a potential marker of pregnancy dating. This study investigated the correlation between the morphometrical skin characteristics with the pregnancy chronology to propose models for predicting gestational age.

**Methods:**

A cross-sectional analysis selected 35 corpses of newborns. The biopsy was performed up to 48 hours after death in the periumbilical abdomen, palm and sole regions. Pregnancy chronology was based on the obstetric ultrasound before 14 weeks. The dimensions of the skin layers, area of glands and connective fibrous tissue were measured with imaging software support. Univariate and multivariate regression models on morphometric values were used to predict gestational age.

**Results:**

Gestational age at birth ranged from 20.3 to 41.2 weeks. Seventy-one skin specimens resulted in the analysis of 1183 digital histological images. The correlation between skin thickness and gestational age was positive and strong in both regions of the body. The highest univariate correlation between gestational age and skin thickness was using the epidermal layer dimensions, in palm (r=0.867, p<0.001). The multivariate modelling with the thickness of the abdominal epidermis, the dermis and the area of the sebaceous glands adjusted had the highest correlation with gestational age (r=0.99, p<0.001).

**Conclusion:**

The thickness of the protective epidermal barrier is, in itself, a potential marker of pregnancy dating. However, sets of values obtained from skin morphometry enhanced the estimation of the gestational age. Such findings may support non-invasive image approaches to estimate pregnancy dating with various clinical applications.

**Summary**

**What is already known?**

- Morphometric invasive analysis of fetal skin provides a visual examination of architectural patterns according to gestational age.
- Non-invasive ultrasound imaging indicates the epidermal thickness of the newborn’s skin as one evolutionary indicator of the gestational chronology.

**What does this paper add?**

- Non-invasive analysis of newborn skin imaging can estimate the dating of pregnancy with various clinical applications.
- The protective epidermal barrier was, in itself, a potential marker of pregnancy dating through skin thickness imaging analysis.
- The multivariate model, including the thickness of the abdominal epidermis, the dermis, and the area of the sebaceous glands, had the highest correlation with gestational age.

## Introduction

The anatomy of the human skin shows a clear relationship between its structure and function.^1^ When well-differentiated, the skin provides a physical and immune barrier essential to newborn survival.^2^ Skin’s barrier function is mainly due to the stratum corneum which is a layer composed of flattened and differentiated corneocytes terminally separated by layers of densely compacted lipides.^1 3^ Studies using skin biopsy are relevant to improve knowledge about the protective barrier during the perinatal period.^4 5^ However, the specimen is difficult to obtain,^6^ and the preparation of slides can result in artefacts and require multiple tissue samples.^6 7^ Even so, microscopic methods with staining procedures allow to outline specific components and measure them in order to portray tissue modifications over time.^8 9^

It is not surprising that the chronology of pregnancy is considered the main indicator of newborn survival.^10^ There are critical clinical relationships between epidermal barrier competence and neonatal survival, faced with the risk of hypothermia and infections.^4^ Histological analysis suggests that epidermal development becomes complete in utero at approximately 34 gestational weeks but will only become functional in the first week of life.^11^ Preterm newborns with gestational age <37 weeks have the thinnest epidermis and a less developed functional barrier than full-term newborns,^12^ being thus poorly prepared to face the extra-utero environment.^11^ These have high rates of water loss and transcutaneous heat loss, in addition to the difficulty in maintaining homeostasis and having a deficient impermeable barrier.^13^

Visible changes in the clinical examination of the newborn’s skin and also in a histological study of this tissue demonstrate that the functional and structural maturation of the skin is a potential marker of the chronology of pregnancy.^14 15^ A non-invasive ultrasound imaging study indicates the thickness of the newborn’s skin as one of the evolutionary indicators that can be objectively measured to estimate the gestational chronology.^7^ In fact, the determining of gestational age with greater accuracy can positively affect perinatal results,^10 16^ as it will direct the most appropriate interventions in neonatal care.^17^ Furthermore, the chronology of gestation is the basis for the statistics of prematurity and nutritional status of the newborn, guiding public policies, which includes the analysis of perinatal mortality.^18^ Nonetheless, the determination of gestational age at birth is not a trivial task since it is directly affected by access to high-cost technology, such as obstetric ultrasound, and by the imprecision of postnatal maturity clinical scores.^19^ New approaches have been proposed, among them the analysis of skin maturity through its optical properties.^20^

This study investigated the correlation between the thickness of the skin layers, area of glands and fibrous connective tissue of the skin in corpses of newborns with the chronology of pregnancy to propose models for predicting gestational age based on morphometry values.

## Methods and materials

### Environment and subjects

Feasibility study evaluated 35 corpses of newborns, stillbirths or dead after birth, prospectively selected in accordance with the eligibility criteria, from January 2016 to September 2019. Based on the expectation of a linear correlation between epidermal thickening and gestational age,^7^ a minimum sample of 17 bodies was calculated to detect a positive and moderate correlation, assuming an alpha error of 5% and one 20% beta error in a two-tailed hypothesis test. They met inclusion criteria as follows: childbirth with gestational age between 20 and 42 weeks of gestation, calculated using the crown-rump length measure ultrasonography-based reference, performed before 14 weeks of gestation.^10^ In the case of stillbirths, the estimated interval between fetal death and childbirth was up to 3 days. For alive newborns selected after decease, the extra uterine life after birth did not exceed 48 hours of age, and biopsy was possible within 24 hours after neonatal death. Exclusion criteria were structural skin alterations or conditions that modify the skin, such as anhydramnios, hydrops, congenital skin diseases and clinical evidence of chorioamnionitis as maternal fever or foul-smelling amniotic fluid; tissue maceration assessed at the visual inspection of the corpses; oedema or autolysis verified during histological analysis.

### The skin biopsy and tissue processing

Human skin specimens were withdrawn from three body regions: over the thenar eminence of palm (palm), over the periumbilical abdominal area and over the calcaneus area (sole of the foot). Punch biopsies cut a circle of 1 cm^2^ of diameter with sufficient depth to reach the full skin thickness and partial hypodermis. The conventional histological preparation included a 10% neutral formalin fixation and 5 µm tissue sections of blocks embedded in paraffin. In addition, the histological slides were stained by Gomori’s trichrome.

### Morphometric analysis of the skin

**Figure 1:**
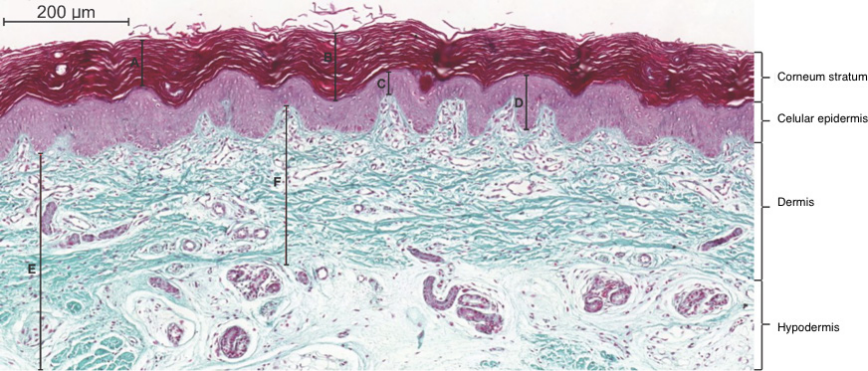
Photomicrograph of the skin on the bottom of the foot of stillbirth at 40 gestational weeks. A represents the measurement of the stratum corneum with a lower limit corresponding to the apex of the epidermal crest. B represents the measurement of the stratum corneum with a lower limit corresponding to the valley of the epidermal papillae. C represents the measurement of the cellular epidermis with a lower limit corresponding to the apex of the dermis. D represents the measurement of the epidermis with a lower limit corresponding to the valley of the epidermal papillae. E represents the measurement of the hypodermis with an upper limit corresponding to the valley of the epidermal papillae. F represents the measure of the upper limit dermis corresponding to the crest of the dermal papilla. Gomori trichrome. Bar=200 mm.

The thickness of the epidermis, dermis, area of the sebaceous and sweat glands were measured, as well as the area of fibrous tissue. A3DHISTECH Pannoramic MIDI (Budapest, Hungary) scanner and Pannoramic Viewer software captured images of the slides. From each slide, 2–5 frames with an objective magnification of ×10 were selected according to image quality criteria, tissue integrity and presence of all skin layers and part of the hypodermis. We set algorithms in the KS300 software of analysis contained in the Carl Zeiss image analyzer (Oberkochen, Germany) to semi-automatically explore the image, based on Caliari procedures.^21^ Epidermal measurements included the thickness of the epidermal layer and the corneum stratum, with the boundary in the image delineated by the observer. The epidermis was identified by its darker colour and stratified keratinocytes, figure 1. Dermal layer thickness corresponded to the measurement from the epidermal–dermal junction to the dermal–hypodermal limits. The average of five smaller and five larger measures were obtained interactively to average represents the thickness and within variance.

A dermal sector with 7.7×10^5^ µm^2^ was obtained by selecting pixels with shades of green, creating a binary image and using digital processing to calculate the dermal fibrous connective tissue area. We set algorithms in the KS300 software of analysis, based on Prata *et al*.^22^ Interactive measurements of each sweat or sebaceous glands were obtained separately, within a dermal and hypodermal sector with 7.27×10^5^ µm^2^, based on procedures described by Costa *et al*.^23^

### Statistical analyses

Descriptive statistics assessed the clinical characteristics of the newborns and skin morphometry variables. Depending on the data distribution, quantitative variables were presented as averages, SDs, medians (minimum and maximum) or IQRs. The coefficient of variation and the 95% CI were calculated by bootstrap to allow inference based on the skin morphometry sample data. Qualitative variables were presented as absolute values and percentages. Univariate and multivariate regression analyses assessed the correlation between gestational age and skin morphometry for each area on the body where skin biopsy was performed. Using the stepwise approach, multiple regression analysis included significant (p<0.05) predictor variables from the univariate models. Durbin-Watson test of residuals evaluated the fit of the models. Coefficient of determination (adjusted R^2^) was carried based on the hypothesis that it was zero. The SPSS V.22.0 was used for the analysis. P values of less than 0.05 were considered to be significant.

## Results

**Figure 2:**
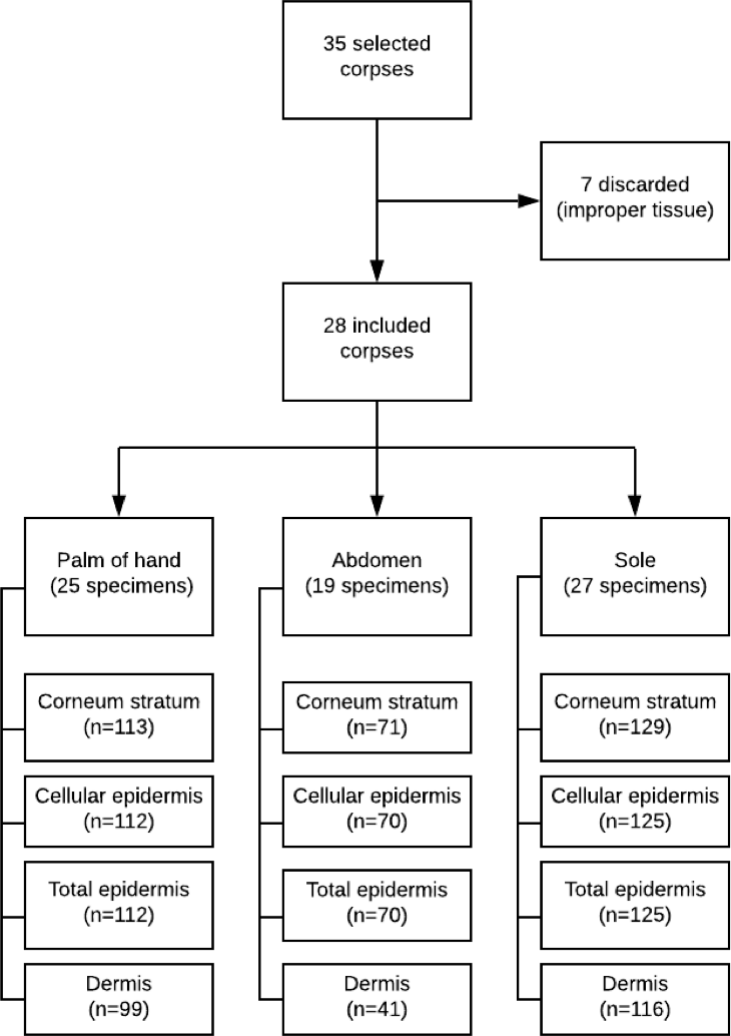
Flowchart diagram detailing the number of analysed images, according to the skin over body areas.

From 35 enlisted corpses, seven did not meet the quality criteria of the skin tissues during histological analysis. Twenty-eight selected newborns gathered 12 (57.14%) after birth and 16 (42.86%) stillbirths. Figure 2 presents details from the enrollment of the newborns to the imagery, according to the assessed segment of the body.

Gestational age ranged from 20.3 to 41.2 weeks of gestation. Clinical characteristics of newborns are described in table 1. The main cause of death was major malformation, accounting for 16 (57.1%—line 3) newborns. There was no difference between stillborn and deaths after childbirth newborns, in relation to the cause of death (p=0.313, line 2), gestational age (p=0.252, line 7), birth weight (p=0.252, line 8), birth weight centile (p=0.840, line 9) and sex (p=0.215, line 10). Among 21 fetuses with gender determination and gestational age at birth equal or above 24 weeks, seven had birth weight below the 10th percentile for gestational age, according to the Intergrowth 21st standard,^24^ three of them stillbirths and four dead after delivery. Two stillbirths had birth weights below the third percentile for gestational age.

**Table 1:** Clinical characteristics of newborns

Characteristics	Stillbirths (n=12)	Dead after delivery (n=16)	P value
Causes of death			0.313*
Major malformation, n (%)	5 (17.86)	11 (39.28)	
Fetal distress, n (%)	2 (7.14)	1 (3.57)	
Diabetes, n (%)	0 (0)	1 (3.57)	
Unknown or others, n (%)	7 (58.3)	3 (18.75)	
Gestational age (weeks), average (SD)	33.1 (17.53)	35.2 (19.8)	0.252†
Birth weight (g), average (SD)	1237.5 (2770)	1935 (3175)	0.252†
Birth weight centile, average (SD)‡	36.1 (39.2)	32.9 (32.6)	0.840†
Sex			0.215*
Male, n (%)	6 (21.43)	3 (10.71)	
Female, n (%)	5 (17.86)	11 (39.28)	
Undetermined, n (%)	1 (3.57)	2 (7.14)	

*χ^2^ test.

†Mann-Whitney test.

‡According to the Intergrowth 21st standard for gestational age ≥24 weeks.^24^

### The thickness of the newborn’s skin layers

One thousand hundred and eighty-three skin images were analysed from 71 slides. The dimensions of the skin layers, their intrinsic variations and comparisons between areas of the body are presented in table 2. The median epidermal thickness on the skin over the palm was similar to that of the sole: 152.1 (43.9–251.9) µm and 146.2 (56.2–276.4) µm (p=0.618), respectively, lines 11 and 12. However, the median thickness of the dermal layer was higher over the periumbilical abdominal area 724.0 (287.0–1107.0) μm, line 16, than sole 396.3 (174.0–493.2) μm, line 15 and palm 384.1 (166.0–751.0) μm, line 14, p<0.001. The standardised variability of measurements for layers of the skin had high value in skin layers over the periumbilical abdominal area, lines 11, 12 and 13.

**Table 2:** Dimensions of the skin layers at birth, with comparisons between the assessed areas of the body

	Median (95% CI)	Min–Max	CV* (%)	Comparisons		
				P value†	P value‡	P value§
Thickness of the corneum stratum (μm)						
Palm	63.6 (21.3 to 81.9)	6.1–154.5	32.9	0.707		
Sole	72.4 (7.6 to 176.0)	7.6–176.0	34.1			0.002
Periumbilical abdominal area	18.0 (8.0 to 43.4)	8.0–43.4	46.6		0.010	
Epidermal thickness (μm)						
Palm	72.0 (33.0 to 101.7)	33.0–101.7	44.2	0.701		
Sole	78.8 (41.2 to 128.5)	41.2–128.5	41.6			<0.001
Periumbilical abdominal area	44.3 (19.0 to 61.2)	19–61.2	41.7		<0.001	
Epidermal total thickness (μm)						
Palm	152.1 (43.9 to 251.9)	43.9–251.9	77.1	0.618		
Sole	146.2 (122.6 to 170.3)	56.2–276.4	74.5			<0.001
Periumbilical abdominal area	66.0 (28.1 to 99.5)	28.1–99.5	85.9		<0.001	
Dermal thickness (μm)						
Palm	384.1 (166.0 to 751.0)	166.0–751.0	21.9	0.977	0.002	<0.001
Sole	396.3 (174.0 to 493.2)	174.0–493.2	20.3			
Periumbilical abdominal area	724.0 (287.0 to 1107.0)	287.0–1107.0	18.7			

*CV: average of the coefficient of variation obtained for each image.

†Difference between palm and sole areas.

‡Difference between palm and periumbilical abdominal area.

§Difference between a sole and periumbilical abdominal area.

The area of fibrous connective tissue of the skin over periumbilical area 0.259×10^6^ µm^2^ (0.093–0.526) had a median value similar to that of the sole 0.235×10^6^ µm^2^ (0.008–0.524) and palm 0.248×10^6^ µm^2^ (0.069–0.346), p=0.708 and p=0.817, respectively (table 3, lines 4, 5 and 6). However, the median value of the area of the sweat glands in the skin over periumbilical area, 0.294×10^6^ µm^2^ (0.020–3.651), was higher than that in the palm 0.097×10^6^ µm^2^ (0.028–0.172) or sole 0.088×10^6^ µm^2^ (0.033–0.242), lines 8 and 9, p<0.001, for both comparisons.

**Table 3:** Concentration of fibrous tissue and glands of the skin at birth, with comparisons between the assessed areas of the body

	Median (Min–Max)	Comparisons		
		P value*	P value†	P value‡
Area of fibrous connective tissue (10^6^ µm^2^)				
Palm	0.248 (0.069–0.346)	1		
Sole	0.235 (0.008–0.524)			0.708
Periumbilical abdominal area	0.259 (0.093–0.526)		0.817	
Area of sweat glands (10^6^ µm^2^)				
Palm	0.097 (0.028–0.173)	0.718		
Sole	0.088 (0.033–0.242)			<0.001
Periumbilical abdominal area	0.025 (0.010–0.061)		<0.001	
Area of sebaceous glands (10^6^ µm^2^)				
Periumbilical abdominal area	0.294 (0.020–3.652)	–	–	–

*Difference between palm and sole.

†Difference between palm and periumbilical area.

‡Difference between sole and periumbilical area.

The correlation between the gestational age and morphometry of the skin at birth is presented in table 4. Scatter plots with the linear correlation of each morphometric variable with the gestational age are in online supplemental file S1 to S13. In the univariate analysis, the epidermal thickness layer highlighted as the dimension strongly associated with gestational age: in the skin over palm (r=0.867, p<0.001, line 3), periumbilical abdominal area (r=0.806, p<0.001, line 8) and sole (r=0.712, p<0.001, line 14). The fibrous connective tissue (lines 5, 10 and 16), sweat or sebaceous glands areas had mild or absent correlations with the gestational age (lines 6, 11, 12 and 17). However, compositions of the morphometric parameters fitted multivariate models better explained the variability of the gestational age than univariate correlations. Considering the skin of the periumbilical area, the composition formed by the thickness of the epidermis, dermis and the area of sebaceous glands showed an excellent correlation with gestational (r=0.99, p<0.001, line 8). Concern the skin over the hand and sole, the multivariate model grouping morphometry parameters also enhanced the model of prediction of gestational age, concerning the univariate models: adjusted r=0.94, p<0.001 (line 3), and r=0.99, p<0.001 (line 8).

10.1136/bmjhci-2021-100476.supp1 Supplementary data

**Table 4:** Predictive models for gestational age, based on morphometry values of the skin at birth

	Univariate analysis	Multivariate analysis	
	Linear coefficient (P value)	Adjusted coefficient of correlation	P value of the model
Skin over palm			
Epidermal thickness (μm)	0.867 (<0.001)	0.655	0.94 (p<0.001)
Dermal thickness (μm)	0.805 (<0.001)	0.256	
Area of fibrous connective tissue (µm^2^)	0.518 (0.014)	0.169	
Area of sweat glands (µm^2^)	−0.143 (0.515)	–	–
Skin of periumbilical abdominal area			
Epidermal thickness (μm)	0.806 (<0.001)	0.559	0.99 (p<0.001)
Dermal thickness (μm)	0.579 (0.038)	−0.216	
Area of fibrous connective tissue (µm^2^)	0.538 (0.071)	–	–
Area of sweat glands (µm^2^)	0.441 (0.131)	–	–
Area of sebaceous glands (µm^2^)	–0.845 (0.001)	−0.646	
Skin over sole			
Epidermal thickness (μm)	0.712 (<0.001)	0.540	0.83 (p<0.001)
Dermal thickness (μm)	0.660 (<0.001)	0.456	
Area of fibrous connective tissue (µm^2^)	−0.266 (0.189)	–	–
Area of sweat glands (µm^2^)	–0.266 (0.189)	–	–

R-square of multivariate models: 0.87 (palm), 0.97 (abdomen), 0.69 (sole). Durbin-Watson analysis: 1.94 (palm), 1.90 (abdomen), 1.45 (sole).

## Discussion

### Main findings

In this study, the main contribution was to correlate dimensions measured by morphometry of the skin of a newborn with its gestational age, a new knowledge that can objectively estimate the chronology of pregnancy from histology. The processing of images and the synthesis of values with inferential statistics on the measurements of layers, sublayers, gland area and fibrous connective tissue allowed the development of mathematical models of prediction. In addition, the study documented the intra-subject variability of these measures, numerically reflecting the ripple of the skin layers, guided by the dermal papillae. Regarding the external validity, the selected sample gathered newborns with a wide range of gestational age from extreme prematurity, 20.3 weeks, to term, 41.6 weeks. Although major malformations were responsible for most deaths (57.1%), conditions associated with changes in skin structure were excluded in the recruitment phase.

Regarding morphometric measurements, the results fill a knowledge gap in the study of human skin in this age group, including samples of premature births. In a systematic review published by De-Souza *et al*,^4^ similar studies that provide measurements of newborn skin thickness were considered insufficient to describe morphometry in a reproducible and detailed manner. In addition to the care with microscopic measurements, the chronology of pregnancy was calculated based on early obstetric ultrasound examination, considered a reference standard for pregnancy dating.^10^

There are numerous challenges of inaccurate calculation of pregnancy chronology by available clinical methods,^19^ and this is also a motivation using of fetal skin histology in pregnancy dating. The proposed models of prediction of gestational age may support the investigation of perinatal death and support non-invasive studies with similar applications.^7 20^ Infant mortality has at preterm birth, one of the major current challenges of obstetric and neonatal care.^19 25^ Although the approach is invasive, using skin biopsy in the corpses of newborns, the process brought an opportunity to estimate the chronology of pregnancy, at the time of death, from the morphometry of the skin of specific regions and technique. The histological analysis of the skin, through the visual analysis of architectural patterns, the tissues already proved predictive of gestational age in a previous study,^26^ without, however, presenting quantitative elements that allow the dating.

### Comparison with prior studies

In relation to the magnitude of the measurements, the thickness of the epidermis was greater in the region of the palm and sole of the foot, in relation to the periumbilical region. This finding confirms previous reports that in these places, the stratification of the epidermis is earlier and more intense than in other regions of the body.^26 27^ The early and progressive multiplication of the epidermis in these places may explain the strong correlation found between the thickness of the skin layers and the chronology of pregnancy, even as an isolated marker. However, the comparability of the values found with previous reports is hampered by the incomplete description of the various measures and techniques already published in the scientific literature. Measurements of part of the sublayers, for example, the thickness of the epidermis without including the stratum corneum, only dermis thickness^9^ and measurements made in different places of the body and ages of the children studied.^27–29^ Besides, the measurement of epidermal thickness, according to Kakasheva-Mazhenkovska *et al,*^30^ was 193.2 µm in the sole of the foot, 161.6 µm in the abdomen and 142.0 µm in the hand, comparable to the present study. The measurements of the epidermis described here also corroborate the findings of a non-invasive study that performed measurements of different sites of the body of newborns through high-frequency ultrasound,^7^ which showed values of the thickness of the epidermis in the region of the sole of the foot were 175.4 (17.6) µm. In the dermal layer, we obtained values apparently lower than 873.0 µm in the palm, 719.9 µm in the sole and 1297.0 µm in the abdomen.^30^ We attribute these differences to variations in technique and gestational age of the samples.

More recently, Dhingra *et al* analysed four regions of the body of 30 fetuses from 11 to 40 weeks of gestation. The epidermal thickness had a significant positive correlation with gestational age.^31^ Our study corroborated such results of a strong correlation with gestational age in the skin over the abdomen and palm. However, this study did not combine variables and nor assess gland area and fibrous connective tissue in the prediction as to the current approach.

### Limitations and highlights

The main limitation of this study was the strict eligibility criteria for pregnancy dating and tissue quality, which made it challenging to obtain the postmortem specimen, considered rare.^32^ On the other hand, we emphasise that the multivariate models achieved high correlation coefficients for groups of morphometric measures, 0.94 in the palm region, 0.99 in the abdomen region and 0.83 in the sole, table 4. In addition, the objective measurement of several tissue components such as the area of connective tissue and glands, to estimate gestational age, is unprecedented. Therefore, the mathematical models have the potential to automate the analysis process and may facilitate in the future the obtaining of gestational age information from the systematised analysis of a histological image of the skin. In addition, we believe that future studies may find utility in the results presented in this analysis in tissue engineering, simulation models of the skin, mainly subsidising more appropriate care with the newborn’s skin.

Besides, seven corpses had birth weight below the 10th percentile for gestational age and two below the third percentile. Even fetal growth reference standards are suboptimal for stillbirths,^33^ the influence of fetal malnutrition in the dimensions of deep layers of the skin is possible. However, the skin surface seems not to be influenced by fetal nutrition. In a prior study, Vitral *et al* analysed 222 alive newborns at birth, with gestational age ranging from 24 to 41 weeks of gestation, using high-frequency ultrasound, and epidermal thickness was not fetal growth standard dependent.^7^

## Conclusions

Skin morphometry, especially the measurement of layer thickness, proved to be an essential marker of gestational age at birth. The representation of structural changes in the skin in composite mathematical models involving various elements of this tissue proved to be promising automating of the pregnancy dating process from histological images.

## Data Availability

Data are available upon reasonable request. Raw data were generated at Universidade Federal de Minas Gerais, Bazil. Derived data supporting the findings of this study are available from the corresponding author ZSNR on request.
