# Supplementary material for: Association between the chronology of gestation and the morphometrical skin characteristics at childbirth: a development of predictive model
Source: BMJ Health Care Inform. 2021 Dec 7;28(1):e100476. doi: 10.1136/bmjhci-2021-100476 (PMC8655593; doi:10.1136/bmjhci-2021-100476)
Supplement: Supplementary data [file bmjhci-2021-100476supp001.pdf]

Supporting information

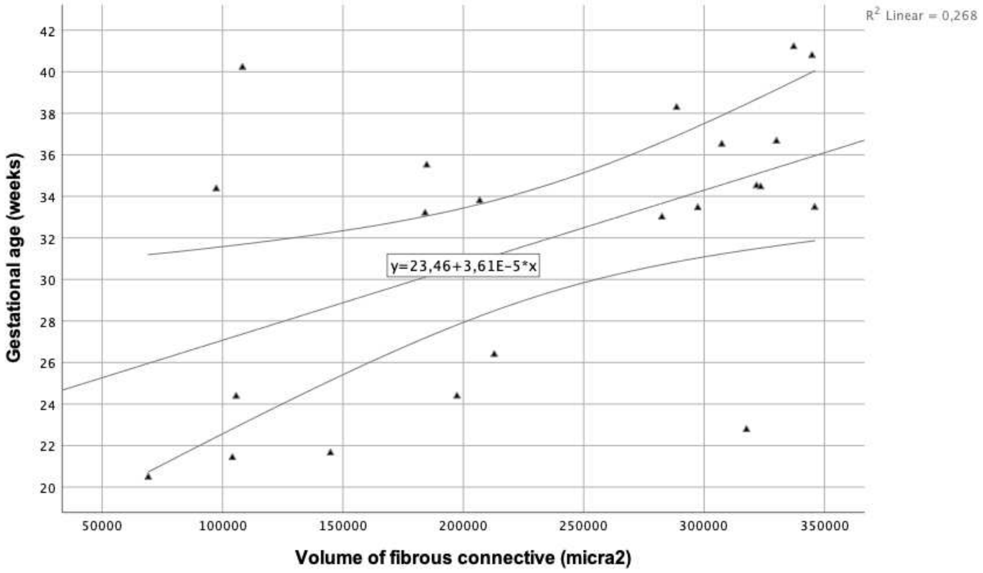

S1 Fig. Univariate analysis of linear correlation between gestational age at birth and the thickness of the epidermal layer over the hand. S1 Fig legend R^2: regression determination coefficient.

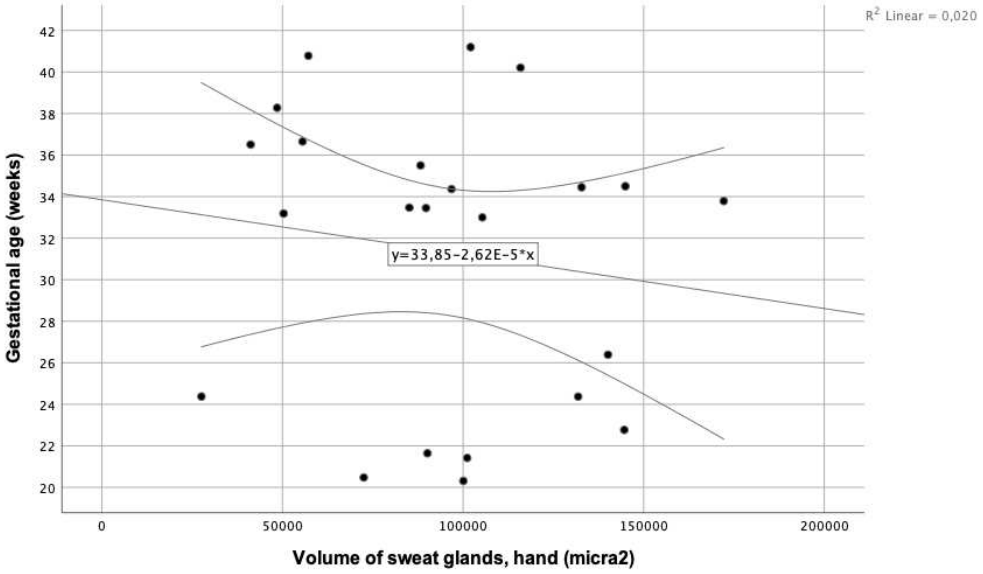

S2 Fig. Univariate analysis of linear correlation between gestational age at birth and the thickness of the dermal layer over the hand. S1 Fig legend R^2: regression determination coefficient.

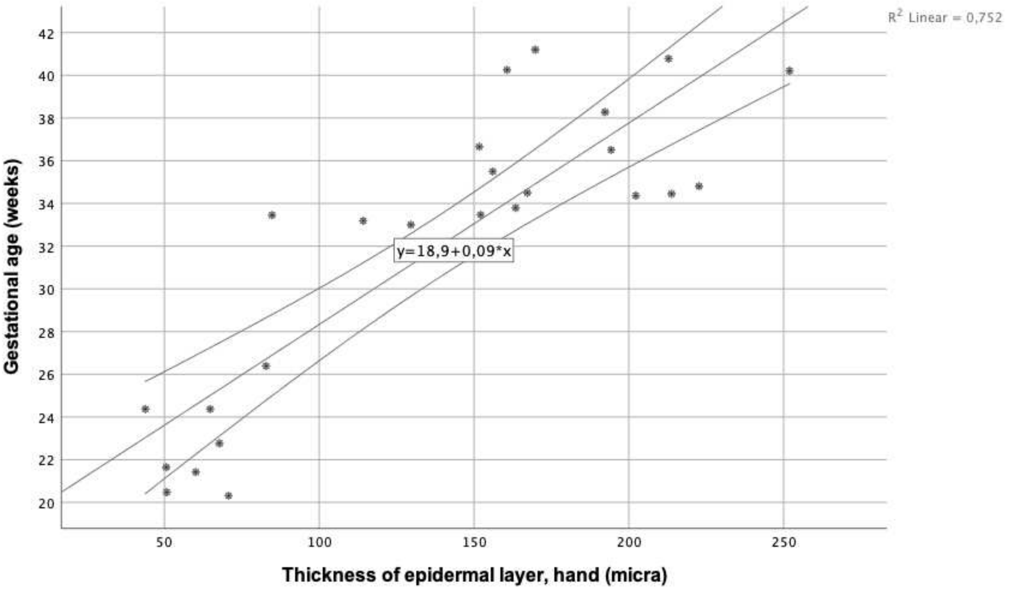

S3 Fig. Univariate analysis of linear correlation between gestational age at birth and the area of the fibrous connective tissue over the hand. S1 Fig legend R^2: regression determination coefficient.

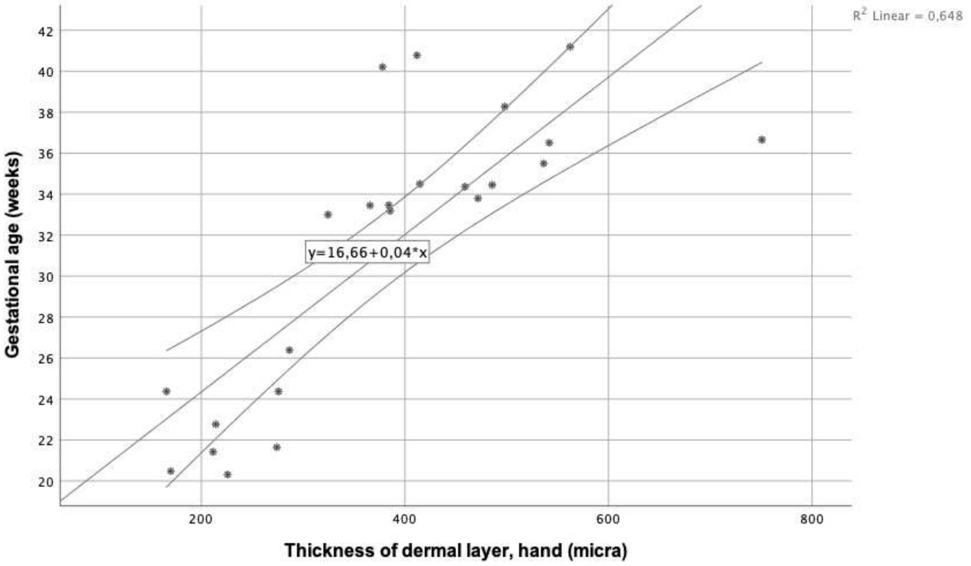

S4 Fig. Univariate analysis of linear correlation between gestational age at birth and the area of sweat glands over the hand. S1 Fig legend R^2: regression determination coefficient.

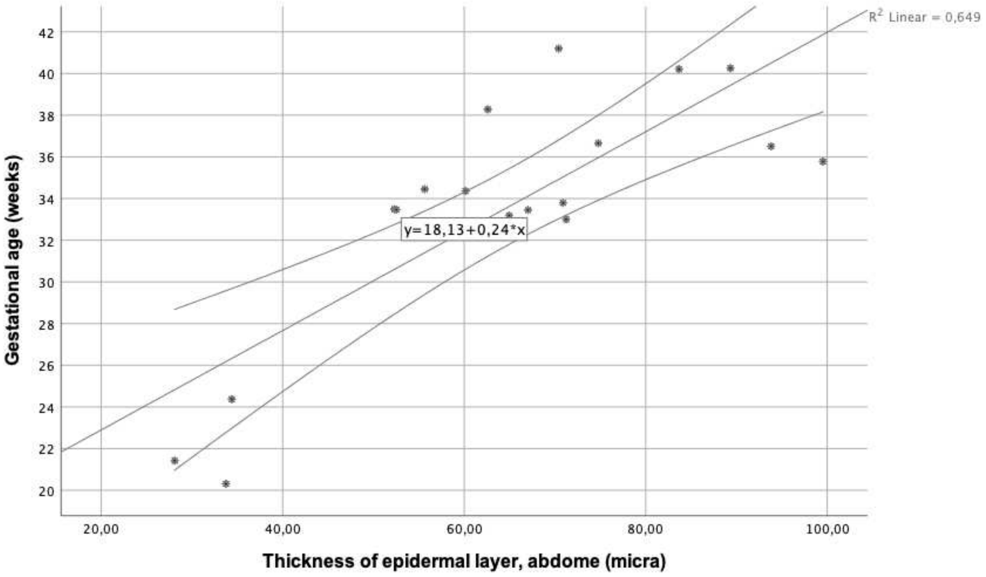

S5 Fig. Univariate analysis of linear correlation between gestational age at birth and the thickness of the epidermal layer over the abdomen. S1 Fig legend R^2: regression determination coefficient.

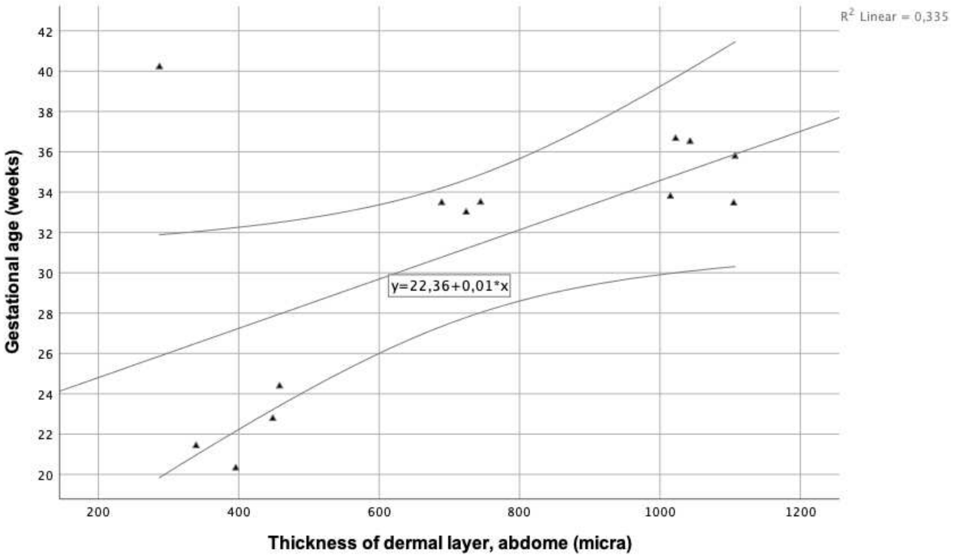

S6 Fig. Univariate analysis of linear correlation between gestational age at birth and the thickness of the dermal layer over the abdomen. S1 Fig legend R^2: regression determination coefficient.

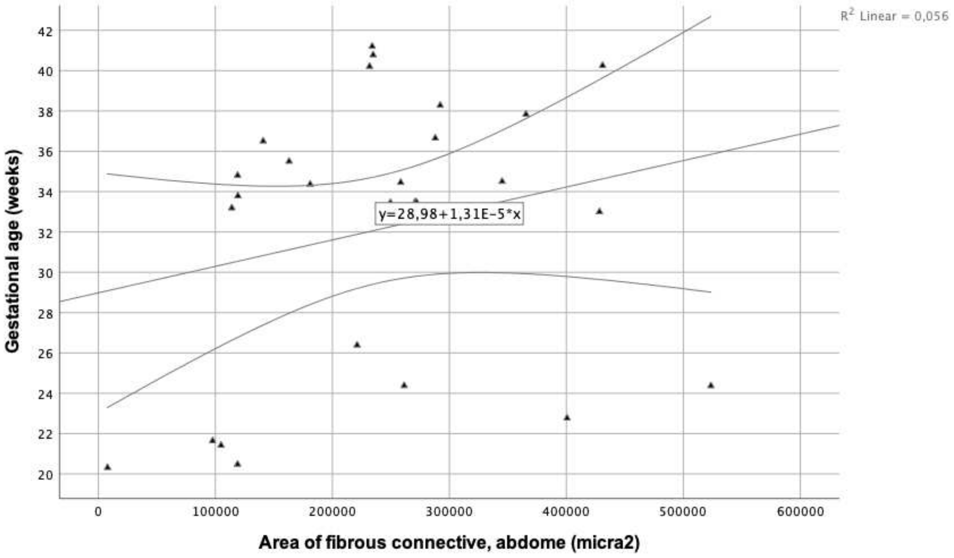

S7 Fig. Univariate analysis of linear correlation between gestational age at birth and the area of the fibrous connective tissue over the abdomen. S1 Fig legend R^2: regression determination coefficient.

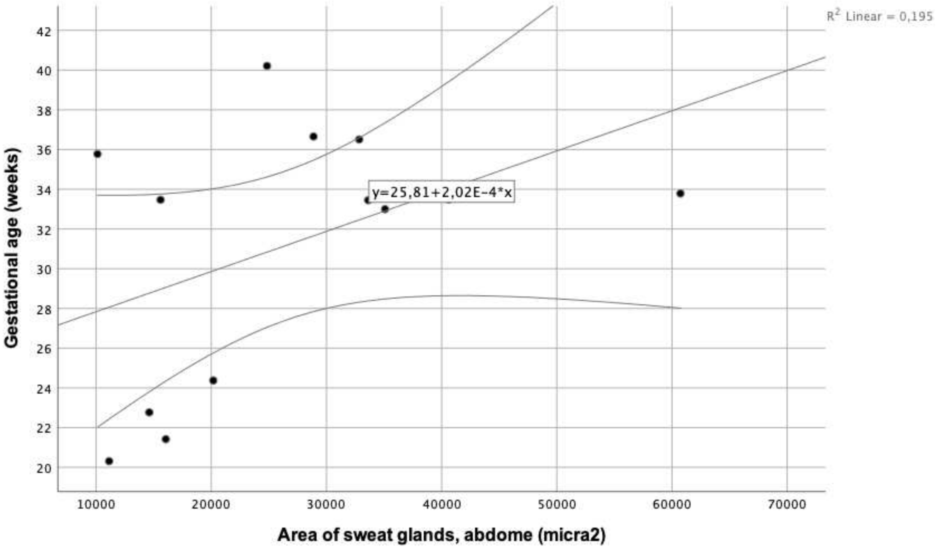

S8 Fig. Univariate analysis of linear correlation between gestational age at birth and the area of sweat glands over the abdomen. S1 Fig legend R^2: regression determination coefficient.

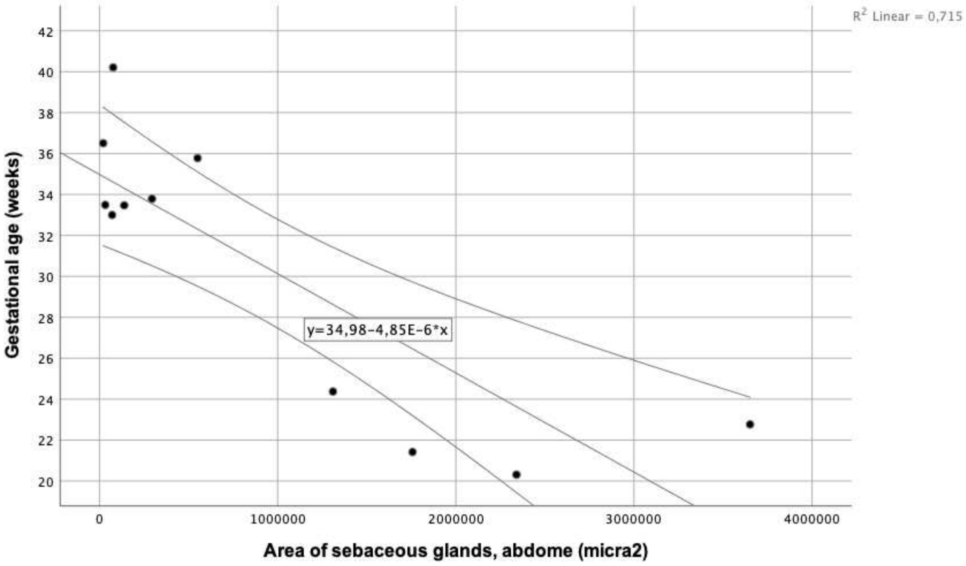

S9 Fig. Univariate analysis of linear correlation between gestational age at birth and the area of sebaceous glands over the abdomen. S1 Fig legend R^2: regression determination coefficient

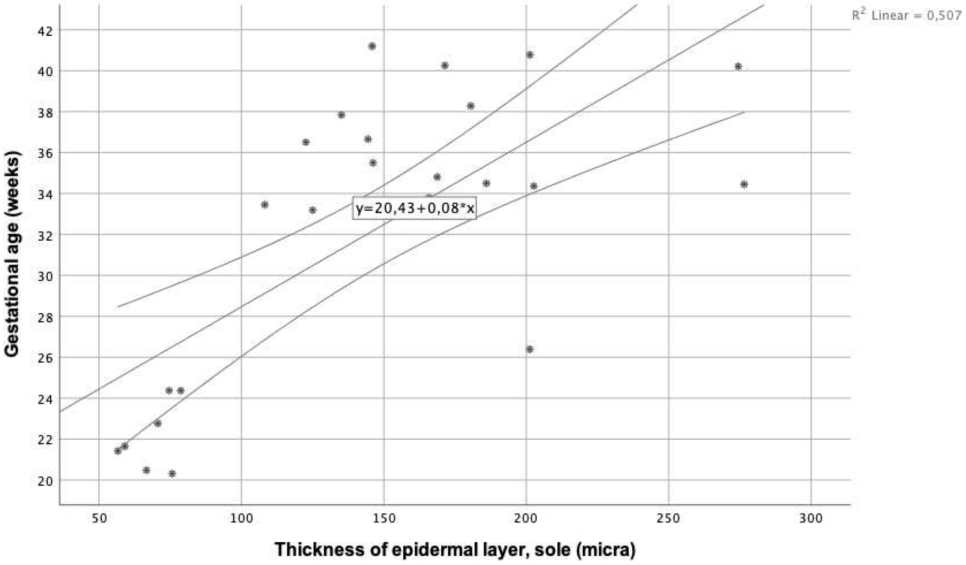

S10 Fig. Univariate analysis of linear correlation between gestational age at birth and the thickness of the epidermal layer over sole. S1 Fig legend R^2: regression determination coefficient.

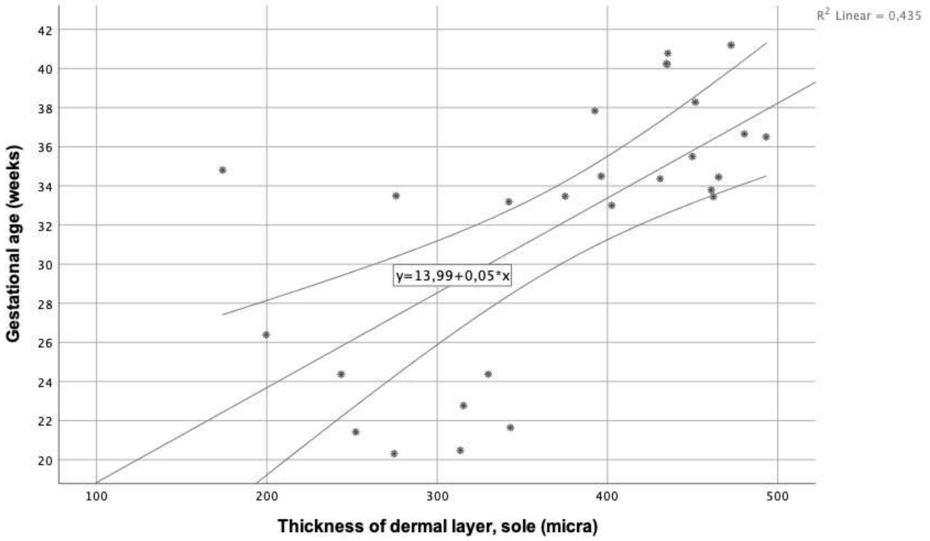

S11 Fig. Univariate analysis of linear correlation between gestational age at birth and the thickness of the dermal layer over sole. S1 Fig legend R^2: regression determination coefficient.

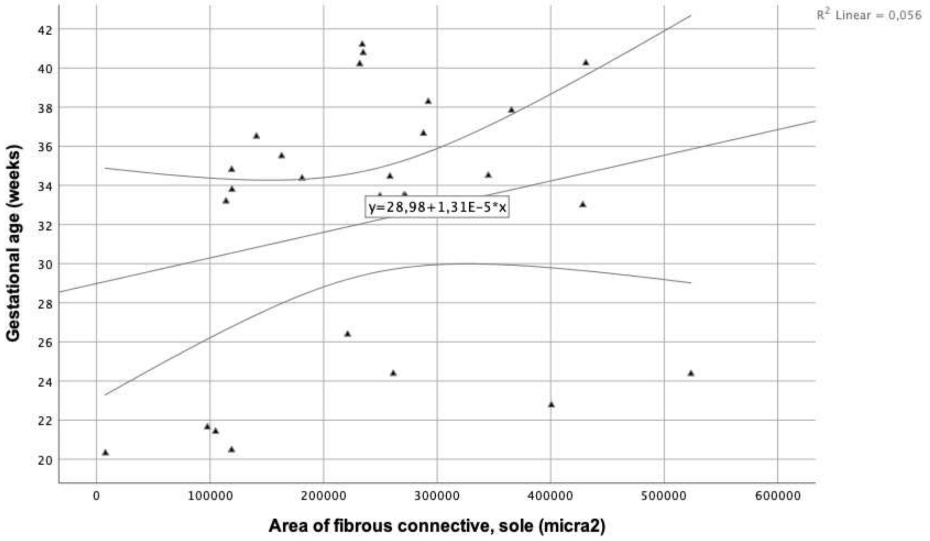

S12 Fig. Univariate analysis of linear correlation between gestational age at birth and the area of fibrous connective tissue over sole. S1 Fig legend R^2: regression determination coefficient.

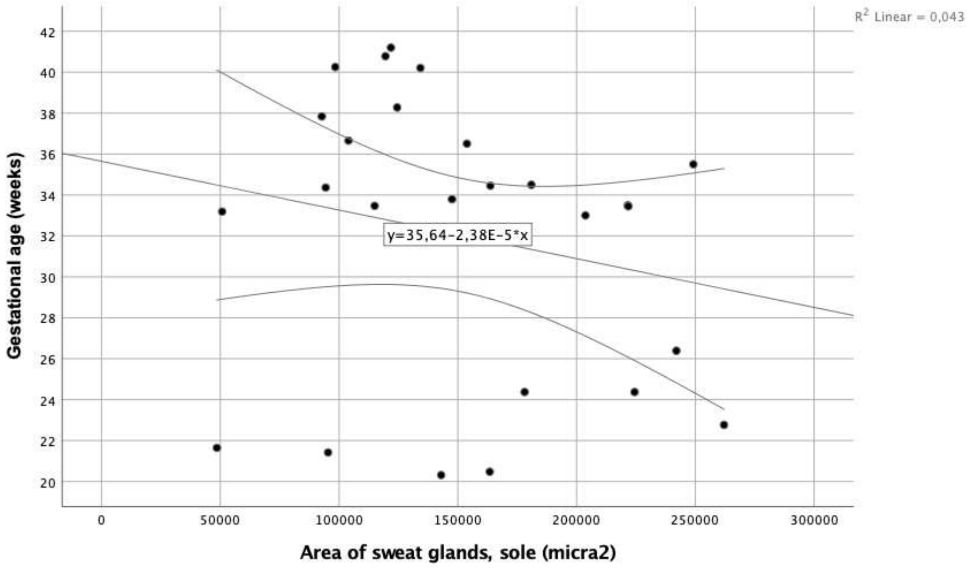

S13 Fig. Univariate analysis of linear correlation between gestational age at birth and the area of sweat glands over sole. S1 Fig legend R^2: regression determination coefficient.
